# Supplementary material for: Seminoma and Embryonal Carcinoma Footprints Identified by Analysis of Integrated Genome-Wide Epigenetic and Expression Profiles of Germ Cell Cancer Cell Lines
Source: PLoS One. 2014 Jun 2;9(6):e98330. doi: 10.1371/journal.pone.0098330 (PMC4041891; doi:10.1371/journal.pone.0098330)
Supplement: File S1 — ZIP file containing DMRforPairs output for significant regions. Please start from the html files. (ZIP) [file pone.0098330.s008.zip › figures/36209.pdf]

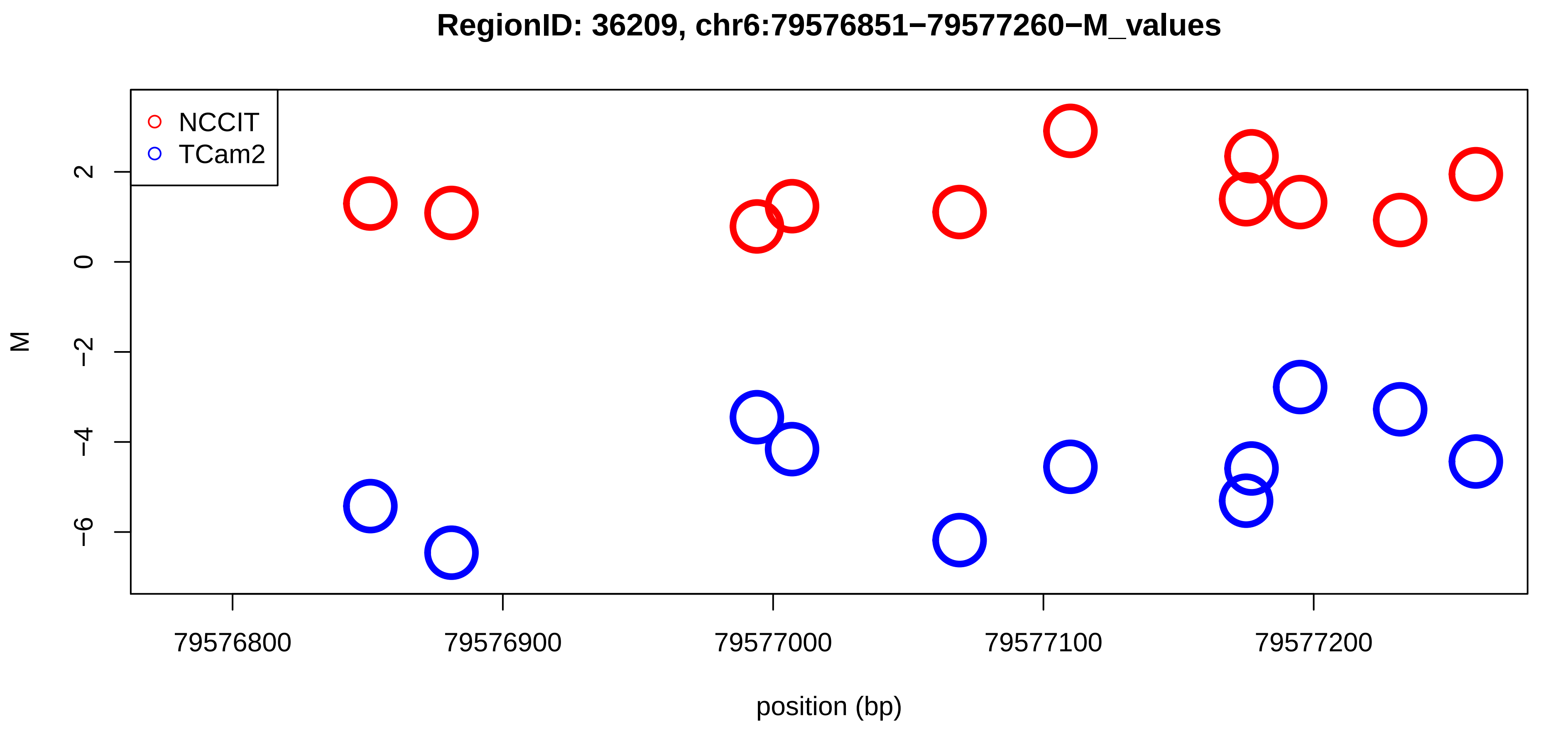
RegionID: 36209, chr6:79576851−79577260−M_values
NCCIT
TCam2
M
2
0
−2
−4
−6
79576800
79576900
79577000
79577100
79577200
position (bp)

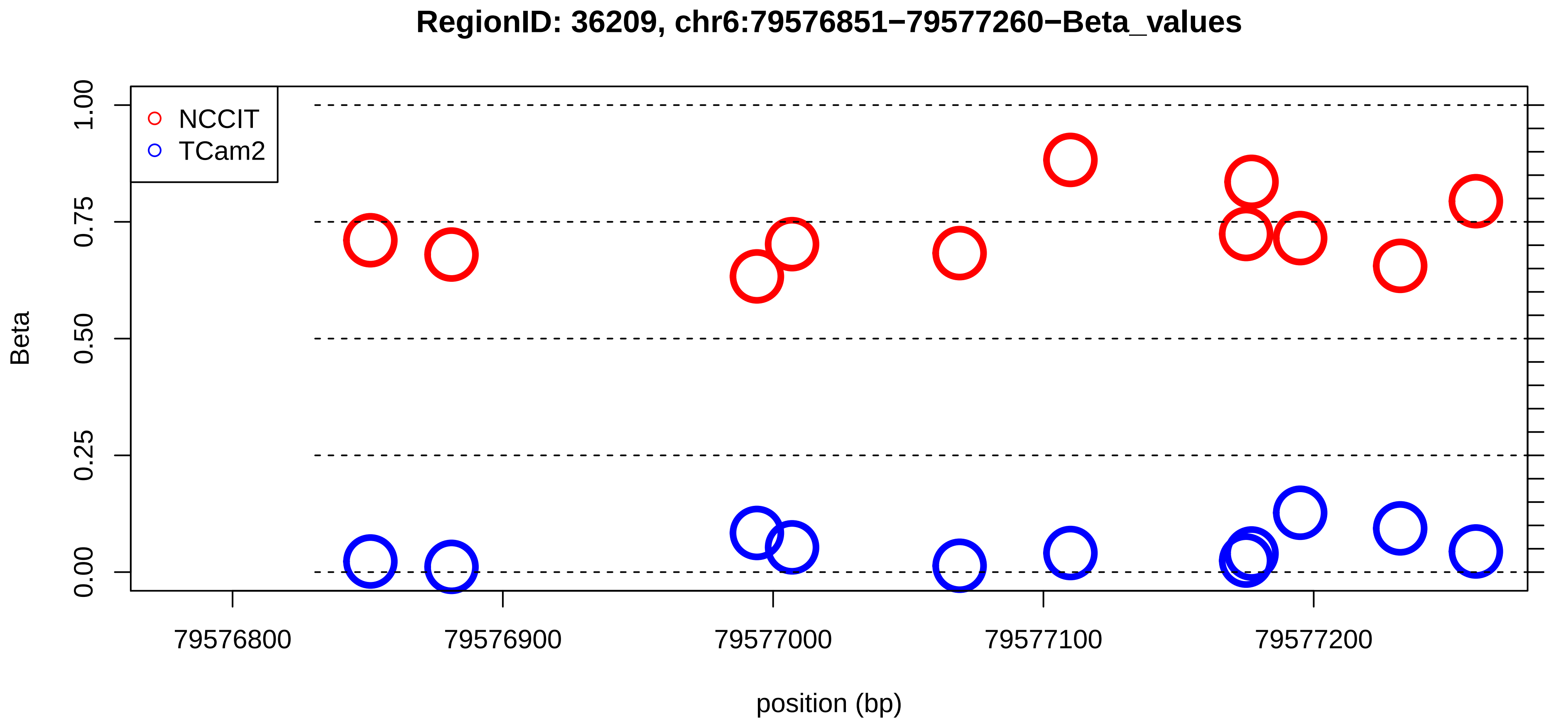
RegionID: 36209, chr6:79576851−79577260−Beta_values
NCCIT
TCam2
Beta
1.00
0.75
0.50
0.25
0.00
79576800
79576900
79577000
79577100
79577200
position (bp)

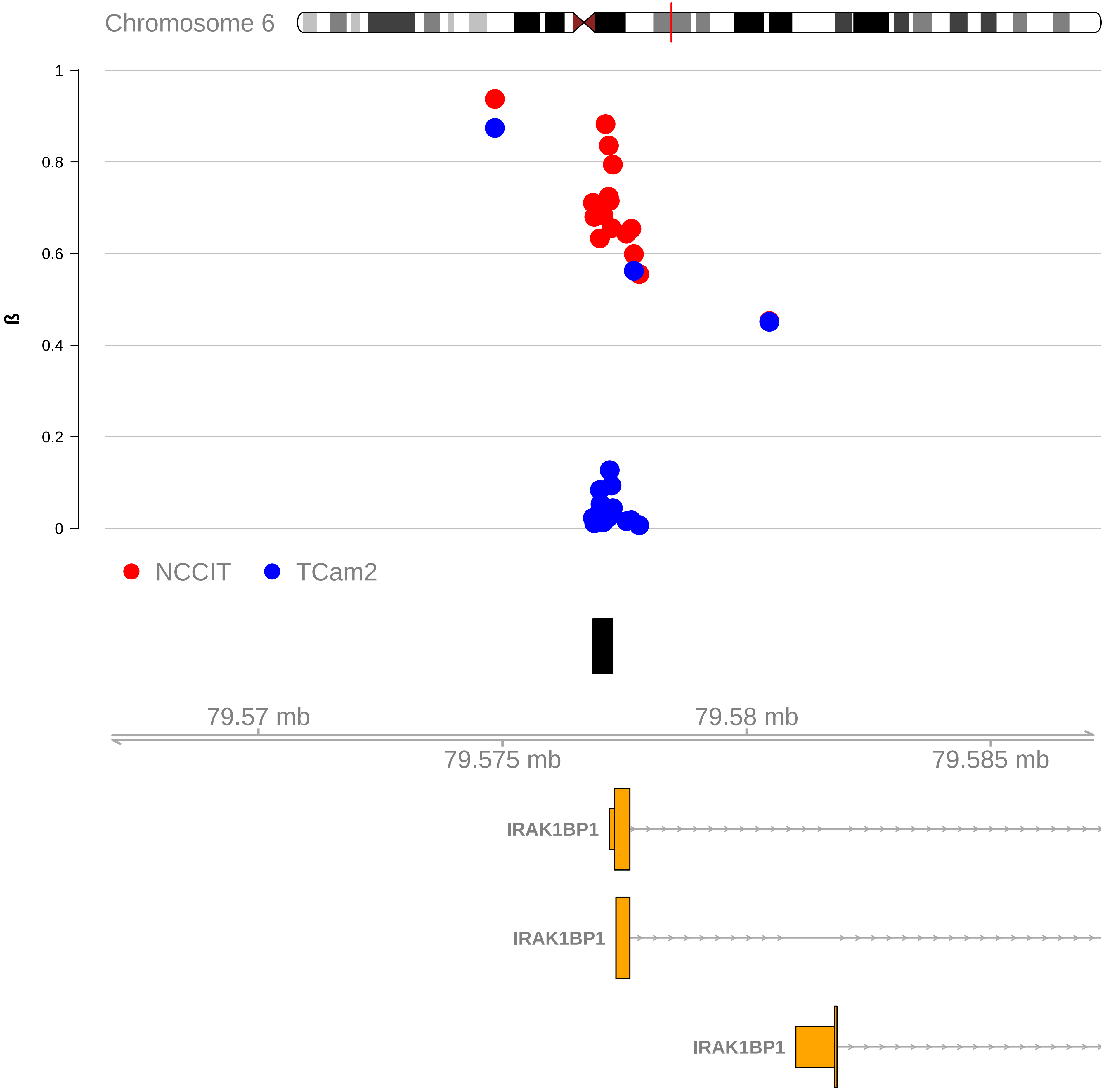

Chromosome 6
1
0.8
0.6
0.4
0.2
0
ß
NCCIT
TCam2
79.57 mb
79.58 mb
79.575 mb
79.585 mb
IRAK1BP1
IRAK1BP1
IRAK1BP1
